# Repeated rectal application of a hyperosmolar lubricant is associated with microbiota shifts but does not affect PrEP drug concentrations: results from a randomized trial in men who have sex with men

**DOI:** 10.1002/jia2.25199

**Published:** 2018-10-31

**Authors:** Richard E Haaland, Jeffrey Fountain, Yingtian Hu, Angela Holder, Chuong Dinh, LaShonda Hall, Nicole A Pescatore, Sheila Heeke, Clyde E Hart, Jiahui Xu, Yi‐Juan Hu, Colleen F Kelley

**Affiliations:** ^1^ Division of HIV/AIDS Prevention Centers for Disease Control and Prevention Atlanta GA USA; ^2^ Department of Biostatistics and Bioinformatics Rollins School of Public Health Emory University Atlanta GA USA; ^3^ Division of Infectious Diseases Department of Medicine The Hope Clinic of the Emory Vaccine Center Emory University School of Medicine Atlanta GA USA; ^4^ Department of Biostatistics St. Jude Children's Research Hospital Memphis TN USA; ^5^ Department of Epidemiology Rollins School of Public Health Emory University Atlanta GA USA

**Keywords:** lubricant, men who have sex with men, pre‐exposure prophylaxis, anti‐retroviral agents, HIV, gut microbiota

## Abstract

**Introduction:**

Oral pre‐exposure prophylaxis (PrEP) with tenofovir disoproxil fumarate (TDF) and emtricitabine (FTC) is highly effective in preventing HIV infection among men who have sex with men (MSM). The effects of consistent personal lubricant use in the rectum on tissue PrEP drug concentrations and the rectal microbiota are unknown. We investigated rectal PrEP drug concentrations and the microbiota in MSM before and after repeated rectal application of a hyperosmolar lubricant.

**Methods:**

We randomized 60 HIV‐negative MSM to apply 4 mL of hyperosmolar rectal lubricant daily (n = 20), take daily oral TDF/FTC (n = 19), or both (n = 21) for seven days. Blood, rectal biopsies and rectal secretions were collected via rigid sigmoidoscopy before and on day 8 after product use. Tenofovir (TFV) and FTC as well as their intracellular metabolites tenofovir‐diphosphate (TFV‐DP), FTC‐triphosphate (FTC‐TP) were measured by HPLC‐mass spectrometry. Rectal mucosal microbiota was sequenced with 16S rRNA sequencing using Illumina MiSeq.

**Results:**

Seven days of lubricant application was not associated with differences in PrEP drug concentrations in rectal tissue or secretions. Lubricant use was associated with a decrease in the relative abundance of the *Bacteroides* genus (*p* =* *0.01) and a non‐significant increase in the *Prevotella* genus (*p* = 0.09) in the rectum. PrEP drug concentrations in rectal tissue and secretions were not associated with microbiota composition or diversity either before or after lubricant use.

**Conclusions:**

Repeated rectal application of a hyperosmolar lubricant does not affect mucosal PrEP drug concentrations but is associated with changes in the rectal microbiome.

## 1 Introduction

Daily oral pre‐exposure prophylaxis (PrEP) with tenofovir disoproxil fumarate (TDF) and emtricitabine (FTC) is highly effective in preventing HIV infection, particularly among men who have sex with men (MSM) 1, 2. Receptive anal intercourse (RAI) remains the primary route of infection for MSM, yet the potential for biological and/or sexual factors to reduce PrEP efficacy remains largely unexplored. Personal lubricants are commonly used by MSM engaging in RAI 3. While lubricants are typically made with ingredients from the “generally recognized as safe” list and do not contain pharmacologically active ingredients, there is a wide range of variability in the composition and chemical properties of personal lubricants 4. In particular, water‐based lubricants with hyperosmolar properties have been shown to induce epithelial tissue damage and cytotoxicity when applied in a single dose to rectal explants and epithelial cell cultures, or applied intermittently to the rectum of rhesus macaques *in vivo* 4, 5, 6, 7, 8, 9. Inflammation caused by tissue damage alters expression of drug metabolizing enzymes and transporters that can affect the absorption, distribution and clearance of antiretroviral (ARV) drugs 10. However, single dose *ex vivo* tissue and intermittent dose *in vivo* animal models did not report lubricant‐associated increases of susceptibility to HIV or SHIV infection 4, 8. In contrast, consistent use of personal lubricants for RAI among a cohort of MSM in the United States was linked to increased acquisition of non‐HIV sexually transmitted infections 11.

Recent reports show that when lubricants were used for nearly 90% of sex acts 3 of MSM engaging in condomless RAI, the rectal mucosal microbiota was enriched for the Prevotellaceae family as compared to Bacteroidaceae for controls. The ratio of *Prevotella* compared to *Bacteroides* in the gut microbiota has received significant attention as these taxa appear to demonstrate an antagonistic relationship and *Prevotella* has been associated with gut markers of inflammation 12, 13, 14. Interestingly, differences in the composition of vaginal mucosal microbiota have been associated with topical PrEP efficacy in the CAPRISA 004 trial 15 but not with oral PrEP efficacy in the Partners PrEP study 16. It is unknown if changes occurring in the rectal mucosa microbiota after repeated use of a hyperosmolar lubricant might affect PrEP drug concentrations in the rectal mucosa. Therefore, we conducted a clinical trial to measure rectal mucosal PrEP drug concentrations and microbiota among MSM who received daily oral TDF/FTC or repeated rectal application of hyperosmolar lubricant, or both in combination.

## 2 Methods

### 2.1 Ethics statement

The study protocol was funded by the US Centers for Disease Control and Prevention (CDC) and approved by Institutional Review Boards at Emory University and CDC and written informed consent was obtained from all study participants. The trial is registered at the ClinicalTrials.gov registry (NCT02401230) and conforms to the US Federal Policy for the Protection of Human Subjects.

### 2.2 Study participants and specimen collection

Sixty HIV‐negative MSM in Atlanta, GA were enrolled in a non‐blinded, randomized clinical trial to apply 4 mL of commercially available, hyperosmolar personal lubricant to the rectum daily with a study‐supplied pre‐filled applicator (n = 20), take oral TDF/FTC (Truvada^®^) daily (Gilead Pharmaceuticals, Foster City, CA) (n = 19), or both (n = 21) for seven consecutive days from March 2015 to July 2016 (Figure S1). Participants were instructed to apply the lube and take TDF/FTC near bedtime at approximately the same time each day and product adherence was evaluated by self‐report. Sample size was estimated to be able to detect a fivefold reduction in tissue drug concentrations among participants using the lubricant. Participants who self‐identified as male aged 19 to 46 years, reported RAI with a man in the last six months, and were in good general health as assessed by the study clinician were eligible to enrol. Men were allocated 1:1:1 to each study arm and randomized in blocks of three by random number generation. Randomization assignments were generated by study staff and concealed in sequentially numbered envelopes until after the study participant provided informed consent and study staff revealed study group assignment.

Participants were asked to refrain from RAI during the intervention, and only men who completed all study visits were included in analyses. The properties of the hyperosmolar lubricant have been described previously (osmolarity: 8064 mmol/kg, pH 4.44) 5. Blood, rectal biopsies, and rectal secretions were collected at two study visits, the first at least 21 days prior to initiating study product and the second on day 8 following seven consecutive days of study product use. Seven days of TDF/FTC is estimated to be sufficient to achieve steady‐state concentrations in rectal mononuclear cells based on a previous study 17. An anorectal swab to test for *Neisseria gonorrhoeae* and *Chlamydia trachomatis* by nucleic acid amplification was collected at each biopsy visit.

Peripheral blood was collected in sodium citrate cell preparation tubes and separated into plasma and peripheral blood mononuclear cell (PBMC) fractions by centrifugation. Biopsies were collected from the mucosa approximately 8 to 10 cm above the external anal aperture, using a rigid sigmoidoscope and flexible sigmoidoscopic forceps (Olympus America, Center Valley, PA) mounted on a semi‐flexible rod. Rectal secretions were collected via rigid sigmoidoscopy using Weckcel spears (Merocel, Mystic, CT). An enema was not used prior to collection of biopsies or rectal secretions.

### 2.3 ARV drug measurement

The primary outcome for this trial was the difference in PrEP drug concentrations with and without lubricant use after seven days of study product use. Laboratory staff members were blinded to study arm assignment. Tenofovir (TFV) and FTC were eluted from Weckcel spears using a previously established method prior to measurement 18. TFV and FTC in rectal fluid eluates were measured by HPLC‐mass spectrometry as previously described with a lower limit of quantification of 10 ng/specimen 19. TFV and FTC concentrations are presented in ng/swab for rectal secretions. Intracellular metabolites tenofovir‐diphosphate (TFV‐DP), emtricitabine‐triphosphate (FTC‐TP) and their corresponding natural substrates, dATP and dCTP in biopsies and PBMCs, were also measured by HPLC‐mass spectrometry as previously described with a lower limit of quantification of 100 fmol/sample (500 fmol/sample for FTC‐TP) for PBMC (5 × 10^6^ PBMC/specimen) and biopsy 20. Intracellular TFV‐DP and FTC‐TP concentrations in PBMC and biopsies were normalized to concentrations per 10^6^ cells and mg tissue weight respectively. Specimens from one participant in the study arm receiving TDF/FTC and lubricant contained no detectable TFV, FTC or their respective intracellular metabolites TFV‐DP or FTC‐TP, suggesting the participant had not taken oral TDF/FTC. This participant was excluded from further analyses.

### 2.4 Microbiome sequencing

Microbiota sequencing before and after study product use was performed as an exploratory outcome for this trial. Rectal mucosal swabs collected approximately 8 to 10 cm from the anal verge were stored in lysis buffer at −80°C. DNA was extracted, amplified and sequenced using methods described previously 21. Microbiomes were unable to be sequenced in specimens collected from one participant in the study arm receiving TDF/FTC and a second participant in the study arm receiving TDF/FTC and lubricant. Reads were trimmed (Q20) and primer sequences removed by means of FastQ‐mcf (https://code.google.com/archive/p/ea-utils/wikis/FastqMcf.wiki). Paired‐end reads were joined using PANDASeq 22 and analysed with QIIME 1.9.1 23. The 16S V1 to V2 contigs were clustered into operational taxonomic units (OTUs) by means of closed‐reference OTU picking at 97% sequence similarity. OTU centroids were aligned using PyNAST 24 and subsequent phylogenetic trees were inferred by FastTree 25. Centroid sequences from each OTU were aligned against the Greengenes database (version 13‐8) for taxonomy assignment 26. Finally, OTUs were summarized at the Genus level, resulting in 340 genera for the following statistical analyses. Raw 16s rRNA sequences were placed in the NCBI Sequence Read Archive under BioProject SRP148821.

### 2.5 Statistical analyses

Distributions of demographics and behavioural characteristics were compared across the three study arms with the non‐parametric Kruskal–Wallis test. Drug concentration distributions were compared between men who took PrEP for seven days and men who took PrEP and used lubricant for seven days with the non‐parametric Wilcoxon rank‐sum test. For microbiome analyses, alpha diversity was measured using the Shannon index. Shannon index and the top 10 most abundant taxa were modelled according to the change in mean diversity and bacterial genus and mean relative abundance before and after study product use across study arms using linear regression models with an indicator for use of lubricant and an indicator for use of PrEP as two covariates with no intercept. We examined associations between the Shannon index and the relative abundance of specific genera of interest, *Bacteroides*,* Prevotella* and *Prevotella_unspecified* before product use and PrEP drug concentrations after product use with the Spearman rank correlation coefficient. Finally, we examined the overall as well as individual influence of all bacterial taxa, including low abundant taxa, measured before study product use on PrEP drug concentrations by applying the linear decomposition model (LDM) method 27 using the Bray–Curtis and Jaccard distance measures; for Jaccard, we rarefied each specimen to 2500 total number of reads (with three out of thirty‐seven specimens collected from men receiving TDF/FTC removed due to insufficient reads), which is sufficient to cover almost full genera based on rarefaction curves, and we obtained multiple rarefied OTU tables to confirm the conclusion. The overall influence of all bacteria was confirmed by the permutational multivariate analysis of variance (PERMANOVA) test 28. We visualized the overall association by the principal coordinates analysis (PCoA) plot (i.e. using the first two principal components of the distance matrix) which coloured the specimens differently for high versus low drug concentration defined as above and below the median.

## 3 Results

Demographic and risk behaviour data for men in the study are reported in Table 1. Median age, race and the number of reported RAI episodes in the last six months were similar between the three study arms.

**Table 1:** Demographic and behavioural characteristics of 60 HIV‐negative MSM enrolled in a three‐arm clinical trial to examine the mucosal effects of lube and PrEP on the rectal mucosal microbiota and PrEP drug concentrations

Characteristic	PrEP only (n = 19)	Lube only (n = 20)	PrEP+Lube (n = 21)	*p*‐value
Median age (range)	28 (22 to 46)	28.5 (19 to 46)	26 (21 to 49)	0.64
Race/ethnicity, n (%)				
White	8 (42)	9 (45)	10 (48)	
Black	9 (47)	11 (55)	11 (52)	0.57
Other	2 (11)	0	0	
Median RAI acts with condom in last 6 months (range)	1 (0 to 26)	1.5 (0 to 20)	2 (1 to 50)	0.87
Median RAI acts without condom in last 6 months (range)	2 (0 to 30)	4 (0 to 30)	5 (1 to 50)	0.07

MSM, men who have sex with men; PrEP, pre‐exposure prophylaxis; RAI, receptive anal intercourse.

**Figure 1:**
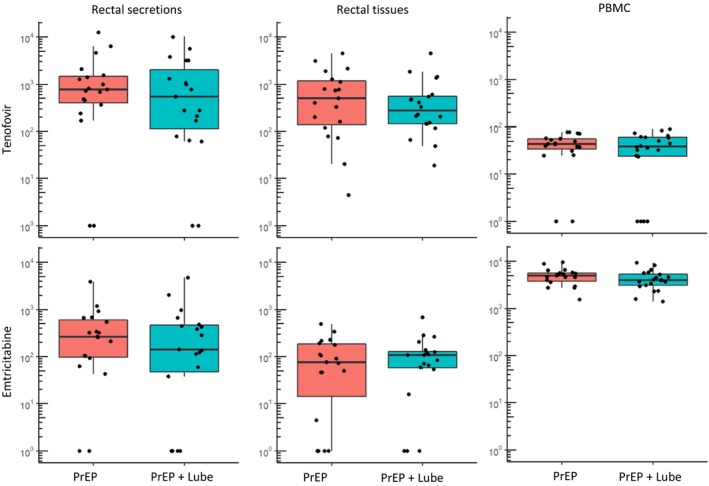
PrEP drug concentrations in rectal secretions, rectal tissue and PBMC in study participants who took oral PrEP stratified by lubricant use. TFV and FTC concentrations were measured by HPLC‐mass spectrometry and are presented in ng/swab for rectal secretions. Intracellular metabolites tenofovir‐diphosphate (TFV‐DP) and emtricitabine‐triphosphate (FTC‐TP) in biopsies and PBMCs were also measured by HPLC‐mass spectrometry and were normalized to concentrations per mg of tissue and per 10^6^ cells respectively. All *p*‐values for median differences between study arms by Wilcoxon rank sum test >0.25. Drug levels below the limit of quantification are displayed at 10°. FTC, emtricitabine; PBMC, peripheral blood mononuclear cell; PrEP, pre‐exposure prophylaxis; TFV, tenofovir; TFV‐DP, tenofovir‐diphosphate.

The proportion of specimens with detectable ARVs in rectal secretions and tissues were similar among men applying a hyperosmolar lubricant (rectal secretions TFV 16/19, FTC 15/19; rectal tissue TFV‐DP 20/20, FTC‐TP 17/20) compared to men receiving TDF/FTC alone (rectal secretions TFV 17/19, FTC 17/19; rectal tissue TFV‐DP 19/19, FTC‐TP 15/19). Specimens with undetectable ARV concentrations were recoded to a value of 0 in subsequent analyses. Concentrations of TFV and FTC in rectal secretions were similar (*p* ≥ 0.25) between men who applied the hyperosmolar lubricant (median, range TFV: 540 ng/swab, 0 to 10,180 ng/swab; FTC: 141 ng/swab, 0 to 4815 ng/swab) and men who did not (median, range TFV: 772 ng/swab, 0 to 12,450 ng/swab; FTC: 261 ng/swab, 0 to 3895 ng/swab) (Figure 1). Similarly, concentrations of TFV‐DP and FTC‐TP detected in rectal tissues were not significantly different among men applying lubricant (median, range TFV‐DP: 280 fmol/mg, 19 to 4536 fmol/mg; FTC‐TP 108 fmol/mg, 0 to 674 fmol/mg) compared to men who did not (median, range TFV‐DP: 510 fmol/mg, 0 to 4488 fmol/mg; FTC‐TP: 75 fmol/mg, 4 to 489 fmol/mg); and no difference was observed in PBMC drug concentrations between men who applied lubricant (median, range TFV‐DP: 38 fmol/10^6^ cells, 0 to 89 fmol/10^6^ cells; FTC‐TP: 4020 fmol/10^6^ cells, 1400 to 9330 fmol/10^6^ cells) and those who did not (median, range TFV‐DP: 44 fmol/10^6^ cells, 0 to 79 fmol/10^6^ cells; FTC‐TP: 4980 fmol/10^6^ cells 1554 to 9470 fmol/10^6^ cells) (Figure 1). One participant who applied lubricant had a rectal *N. gonorrhoeae* infection at the time of specimen collection, but TFV (170 ng/swab) and FTC (141 ng/swab) in rectal secretions as well as TFV‐DP (151 fmol/mg) and FTC‐TP (65 fmol/mg) in rectal tissues remained within the range of concentrations observed in other study participants who applied lubricant. Median TFV‐DP:dATP and FTC‐TP:dCTP ratios were similar among men who applied the hyperosmolar lubricant (TFV‐DP:dATP 5.1; FTC‐TP:dCTP 1.4) when compared to those who did not (TFV‐DP:dATP 4.4; FTC‐TP:dCTP 2.1). Intracellular concentrations of dATP and dCTP in rectal tissues remained unchanged following lubricant application (data not shown).

**Figure 2:**
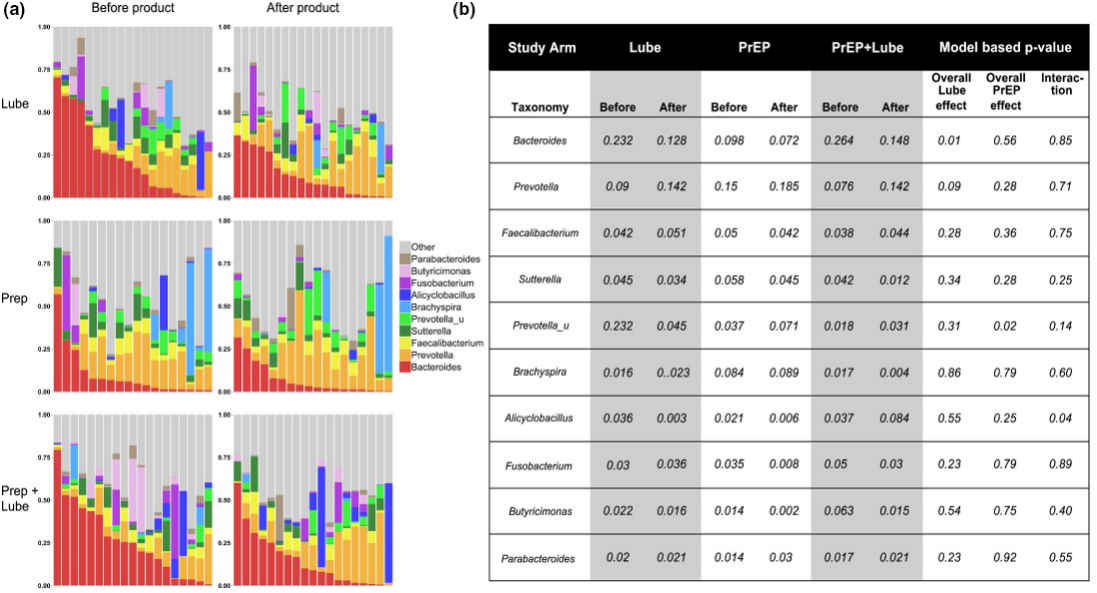
Lubricant use is associated with shifts in rectal microbiota composition. Mean relative abundance of the top 10 most abundant genera in the rectal mucosa before and after study product use stratified by study arm: Lube only, PrEP only, or PrEP and Lube use for seven days (**a**). Change in mean relative abundance before and after study product use in terms of lubricant and PrEP use for the top 10 genera were examined by linear regression modelling (**b**). PrEP, pre‐exposure prophylaxis.

Summary data for the relative abundance of the top 10 genera across the three study arms and between study visits are presented in Figure 2. Overall, there was a significant (*p *=* *0.01) decrease in the relative abundance of *Bacteroides* and a non‐significant (*p *=* *0.086) increase in the relative abundance of the *Prevotella* genus associated with lubricant use. Mean *Bacteroides* abundance in the lube arms before study product use was 23.2% before and 12.8% after study product use (Figure 2b). Mean *Prevotella* abundance in the lube arms before study product use was 9% before and 14.25% after study product use. There were no differences in less abundant taxa associated with lubricant use. Lubricant use was also associated with an increase in microbiota diversity as measured by Shannon index (mean Shannon index before product = 2.44 vs. after product = 2.78; *p *=* *0.006). TDF/FTC use was associated with a small, but significant, increase in the mean relative abundance of an unspecified genus of Prevotellaceae (mean before 3.7% vs. after 7.1%; *p *=* *0.02), but no other changes in the composition or diversity of the microbiota were associated with TDF/FTC use. There was evidence (*p *=* *0.039) for statistical interaction between oral PrEP and repeated application of lubricant only for the relative abundance of the genus *Alicyclobacillus*.

**Figure 3:**
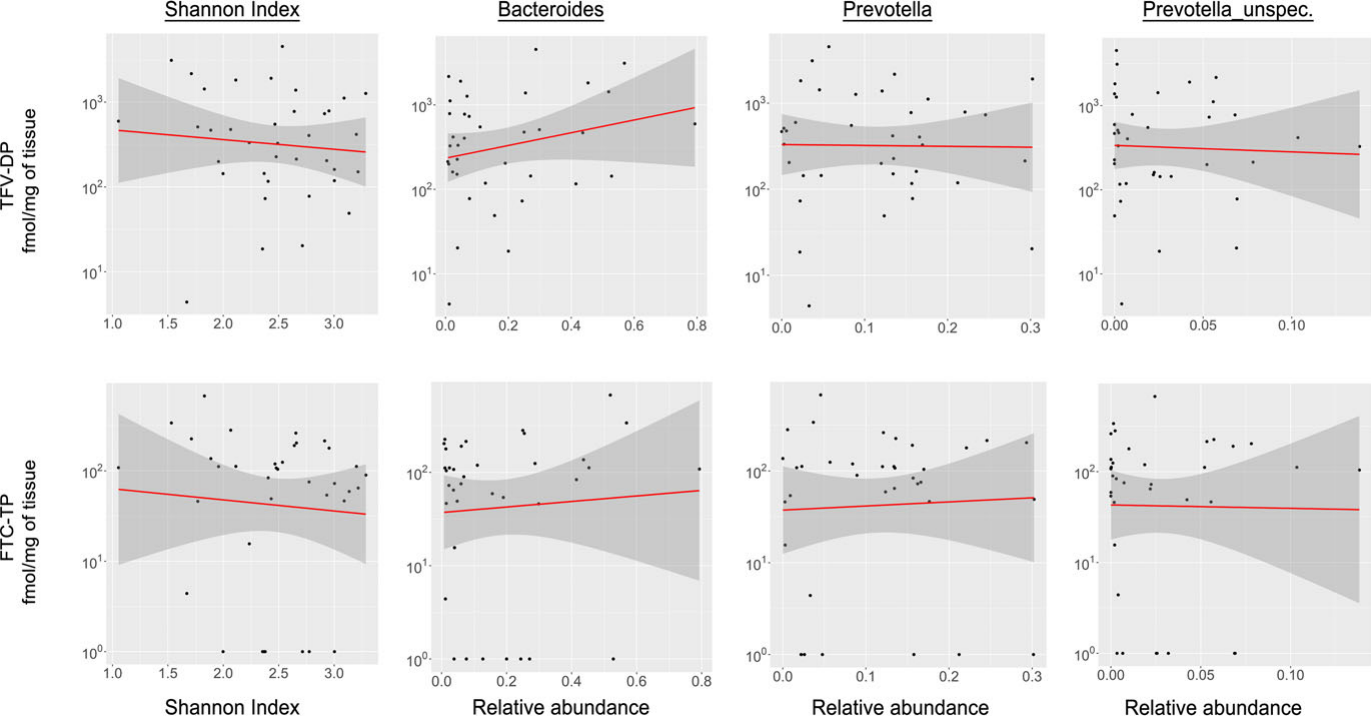
Microbiota species are not associated with differences in tissue PrEP drug concentrations. Associations by Spearman correlation between gut microbial diversity (Shannon index), the relative abundance of *Bacteroides*,* Prevotella* or an unspecified genus of Prevotellaceae as measured before study product use, and PrEP drug concentrations in rectal tissues following seven days of oral TDF/FTC dosing. All *p*‐values for correlations >0.25. Similar results were seen for PBMC and rectal secretions specimens. FTC, emtricitabine; PBMC, peripheral blood mononuclear cell; PrEP, pre‐exposure prophylaxis; TDF, tenofovir disoproxil fumarate.

**Figure 4:**
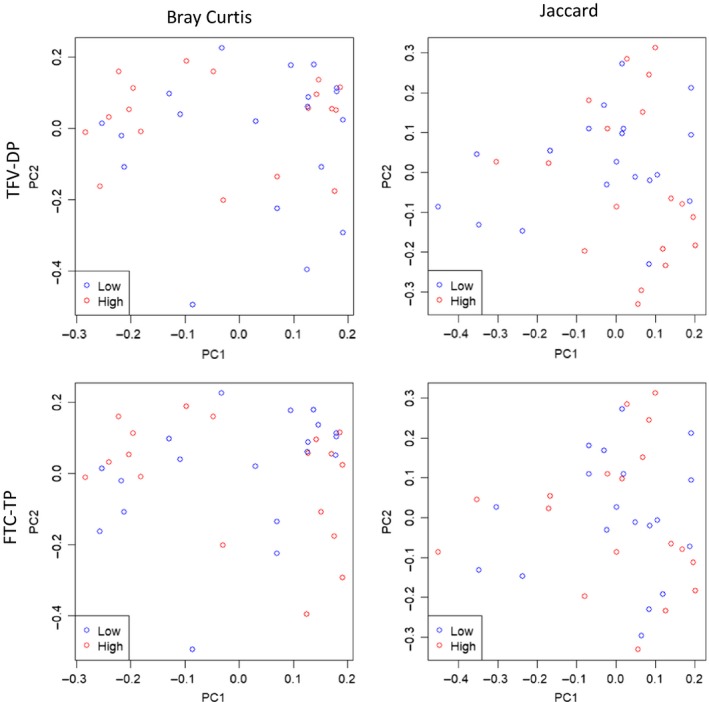
Microbiota composition are not associated with changes in PrEP drug concentrations. Representative data for TFV‐DP and FTC‐TP drug concentrations in rectal tissues following seven days of oral TDF/FTC dosing were stratified at the median into low (blue circles) and high (red circles) drug level groups. Bray–Curtis and Jaccard distance‐based principal coordinates analysis (PCoA) plots are shown for rectal tissues demonstrating no separation between specimens with low and high drug levels. Similar results were seen for PBMC and rectal secretions specimens. FTC, emtricitabine; PBMC, peripheral blood mononuclear cell; PrEP, pre‐exposure prophylaxis; TDF, tenofovir disoproxil fumarate; TFV, tenofovir; TFV‐DP, tenofovir‐diphosphate.

Finally, we examined the effect of microbiota diversity and composition on TDF/FTC drug concentrations in PBMCs, rectal secretions and rectal tissue (representative data shown for rectal tissue in Figure 3). We found no significant associations between the Shannon index, relative abundance of *Bacteroides* or relative abundance of *Prevotella* (including an unspecified genus of Prevotellaceae), measured before study product use with TDF or FTC drug concentrations in biopsies or secretions after product use. To examine if all taxa, including lower abundant taxa, were overall associated with differences in PrEP drug concentrations, PCoA plots using the Bray–Curtis and Jaccard distance measures were constructed and did not show separation between high and low drug concentrations (representative data for rectal tissue shown in Figure 4). When considering drug concentrations as continuous variables, LDM generated a near statistically significant (*p *=* *0.053) global association using Bray–Curtis but detected no associated genera, while PERMANOVA yielded a slightly larger *p*‐value (0.056), suggesting a possible association between the overall composition of the microbiota and PBMC FTC‐TP drug concentrations, but no other drug concentrations examined. Both LDM and PERMANOVA generated non‐significant global associations using Jaccard, indicating no association between the overall presence–absence of the microbiota and any drug concentration.

## 4 Discussion

Hyperosmolar lubricants are commonly used during RAI, but their use may exacerbate tissue damage during RAI raising concerns that cytotoxic effects could potentially reduce the efficacy of biomedical interventions to prevent HIV. This study examined potential changes in the rectal mucosal microbiota and PrEP drug concentrations that may occur with repeated rectal application of a representative product of hyperosmolar water‐based personal lubricants. The results presented here indicate that despite alterations in microbiota diversity and composition associated with repeated rectal application of hyperosmolar lubricant, PrEP drug concentrations in blood, rectal secretions and tissue remain similar among MSM who apply lubricant and those who do not.

Studies in non‐human primates and tissue explants indicate cytotoxic effects of hyperosmolar lubricants do not increase susceptibility to SHIV or HIV infection respectively 4, 8. These results combined with the observation that lubricant application does not significantly affect the concentration and metabolism of PrEP ARVs indicate ARVs are likely to retain their protective efficacy even as lubricants are used during RAI. Certain personal lubricants may also contain anti‐HIV properties as has been suggested previously 29, 30. Furthermore, systemic drug concentrations predict protection from oral dosing regimens suggesting that despite localized tissue damage caused by hyperosmolar personal lubricants, systemic drug concentrations will remain protective in persons adherent to daily oral PrEP 31, 32. These observations are consistent with results of clinical trials indicating daily TDF/FTC remains highly protective among adherent study participants even in the presence of additional biological and behavioural factors 1, 2.

The concentrations of TFV‐DP and FTC‐TP in rectal tissue as well as TFV and FTC in rectal secretions presented here are within ranges of previously reported tissue concentrations among MSM receiving oral TDF/FTC for PrEP 33. The maintenance of intracellular ARV metabolite concentrations in rectal tissue and extracellular ARV concentrations in rectal secretions indicates HIV target cells within mucosal tissues are likely to maintain ARV concentrations consistent with protection even in the presence of tissue damage caused by hyperosmolar lubricants. Hyperosmolar lubricant properties could induce transient release of drug‐containing fluid from tissues. However, as lubricant application and oral TDF/FTC dosing were concurrent, it is likely the lube mucosal effect occurred prior to oral drug reaching rectal tissue resulting in no differences in drug measured in rectal secretions. However, repeated lubricant application is likely to induce tissue damage and inflammation resulting in detectable differences in intracellular drug metabolites that accumulate with subsequent dosing, yet we were unable to detect such differences in this study. TFV‐DP and FTC‐TP are natural analogs of dATP and dCTP, respectively, and decreased ratios of TFV‐DP to dATP have been associated with cellular activation 34, 35, 36. However, immune activation and metabolic changes associated with inflammation did not result in a corresponding decrease in the ratio of either TFV‐DP:dATP or FTC‐TP:dCTP, which might have resulted in reduced efficacy.

While expression of drug metabolizing enzymes and transporters is altered during inflammation 10, our results suggest these changes may not affect ARV concentrations in mucosal tissues. It is possible that lubricant use insignificantly reduced mucosal ARV concentrations due to the number of study participants in this study. However, it is also possible that adherence to daily oral PrEP and the ability of the rectum to repair itself rapidly combine and continually replenish drug concentrations in rectal tissues even in the presence of short‐lived tissue damage. It is notable that imperfect adherence to daily oral dosing PrEP regimens continues to challenge implementation efforts and our study was not designed to determine if lubricant use may exacerbate suboptimal drug concentrations in these situations.

Recent data on suboptimal efficacy of topically applied TFV in the setting of dysbiosis in the female genital tract have underlined the importance of potential interactions between the mucosal immune environment and biomedical HIV prevention interventions 15. We showed an enrichment for *Prevotella* over *Bacteroides* in the rectal mucosa with repeated application of hyperosmolar lubricant. Previous studies, including one from members of our group, demonstrated enrichment of the rectal microbiota for *Prevotella* among MSM, who commonly use lubricants for RAI, and these data suggest an important mechanism for this finding 37, 38. We hypothesize *Prevotella* may be better able to metabolize products of mucosal injury, such as may occur with repeated application of hyperosmolar lubricant, and thus flourishes in this environment. Other factors such as microtrauma during intercourse, semen exposure or enema use may also cause mucosal injury, and contribute to *Prevotella* enrichment among MSM and deserve further investigation.

Nonetheless, it is still unclear that enrichment for *Prevotella* in the gut microbiota represents a pathogenic or “dysbiotic” state. *Prevotella* and *Bacteroides* both belong to the Bacteroidetes phylum, which contributes to a healthy gut microbiota; however, some have suggested enrichment for *Prevotella* versus *Bacteroides* represents a distinct “enterotype” 39, or underlying microbial community, while others have not confirmed this finding 40, 41. *Prevotella* has been associated with gut mucosal markers of inflammation in chronic HIV infection 13, rheumatoid arthritis and bacterial vaginosis 14, although some associations are likely confounded by sexual behaviour. However, *in vitro* data do not support a clear mechanism for a direct inflammatory response to *Prevotella*, and its association with plant‐based diets suggests that enrichment for *Prevotella* alone is not, by itself, pathogenic 42, 43. While further research will certainly be needed to elucidate the relationship between *Prevotella* and mucosal inflammation, our data show there is no association of gut microbiota diversity or composition, including both majority and low abundant taxa, with mucosal PrEP drug concentrations after oral dosing. This reassuring finding is consistent with the preservation of oral PrEP efficacy seen in the setting of vaginal dysbiosis in the Partners PrEP study 16. While our data do not support a reduction in PrEP drug concentrations with differences in the gut microbiota when dosed orally, it is still unknown whether gut microbiota diversity or composition can influence rectal mucosal HIV transmission or efficacy of other biomedical prevention interventions such as candidate rectal microbicides or vaccines and deserves further investigation.

This study has a number of limitations as it was designed to model the effects of repeat rectal application of a hyperosmolar lubricant on mucosal PrEP drug concentrations. Microbiome analyses were initially planned as future, exploratory topics; however, the recent data from the female genital tract prompted us to prioritize these analyses. Lubricant application was documented by self‐report and some men may not have applied the lubricant; nonetheless, the finding of shifts in the microbiota in the lubricant study arms suggests adherence to study product. Additionally, the changes in the microbiota are assumed to result from tissue damage and inflammation caused by the lubricant; however, these direct effects of the lubricant were not tested for in this study. The small sample size and the wide range of tissue ARV concentrations may obscure subtle changes in ARV concentrations following lubricant application, and statistical power was insufficient to detect small differences in bacterial taxa abundance with study product use. This study modelled the effects of hyperosmolar lubricants on PrEP drug concentrations in the rectal mucosa in the absence of RAI. It is unclear if the combination of coitus and lubricant application might create unfavourable changes in the protection afforded by daily oral PrEP. Additionally, the lubricant studied here is one of a number of commercially available lubricants and was chosen as a representative of water‐based hyperosmolar personal lubricants; therefore, the results presented here may not be applicable to other products.

## 5 Conclusions

Repeated application of a hyperosmolar lubricant did not affect mucosal PrEP drug concentrations among MSM, but was associated with changes in the rectal microbiome. While hyperosmolar personal lubricants may exacerbate damage to the rectal mucosa during RAI and lead to shifts in the rectal mucosal microbiota to favour *Prevotella* over *Bacteroides*, protective mucosal and systemic drug concentrations in persons adherent to daily oral TDF/FTC remain unchanged and are likely to retain the ability to prevent HIV infection among MSM.

## Competing interests

The authors declare they have no competing interests.

## Authors’ contributions

REH, CEH and CFK designed the research study. LH, NAP and SH recruited study participants and collected data and specimens for analysis. JF, AH and CD performed and analysed drug measurements. YH, YX and Y‐J H performed and analysed microbiome data. REH and CFK wrote the manuscript with contributions and interpretation of findings from all co‐authors. All authors have read and approved the final manuscript.

## Supporting information


**Figure S1.** CONSORT flow diagram of study participants.
