# Supplementary material for: Repeated rectal application of a hyperosmolar lubricant is associated with microbiota shifts but does not affect PrEP drug concentrations: results from a randomized trial in men who have sex with men
Source: J Int AIDS Soc. 2018 Oct 31;21(10):e25199. doi: 10.1002/jia2.25199 (PMC6207839; doi:10.1002/jia2.25199)
Supplement: Supplementary file 1 — Figure S1. CONSORT flow diagram of study participants. [file JIA2-21-e25199-s001.doc]

**Figure S1. CONSORT flow diagram of study participants**

**Enrollment**

Excluded (n=25)

♦  Not meeting inclusion criteria (n=8)

♦  Declined to participate (n=9)

♦  Other reasons (n=8)

Analysed (n=19)
♦ Excluded from analysis (give reasons) (n=0)

Lost to follow-up (n=2): *no response*

Discontinued intervention (n=1): *unrelated hospitalization*

Analysed (n=21)
♦ Excluded from analysis (give reasons) (n=1) *not adherent*

Lost to follow-up (n=2): *no response*

Discontinued intervention (n=0)

Analysed (n=20)
♦ Excluded from analysis (give reasons) (n=0)

Lost to follow-up (n=3): *scheduling, changed mind*

Discontinued intervention (n=2): *unrelated AE, changed mind*

**PrEP/Lube**

Assessed for eligibility (n=101)

Randomized (n=76)

**Allocation**

**PrEP**

**Lube**

Allocated to intervention (n=25)

♦ Received allocated intervention (n=22)

♦ Did not receive allocated intervention (n=3): *no show, scheduling*

Allocated to intervention (n=25)

♦ Received allocated intervention (n=23)

♦ Did not receive allocated intervention (n=2): *changed mind, no show*

Allocated to intervention (n=26)

♦ Received allocated intervention (n=25)

♦ Did not receive allocated intervention (n=1): *scheduling*

**Follow-Up**

**Analysis**
